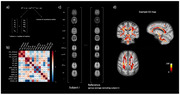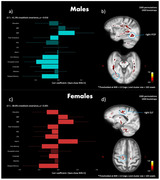# Links between cognition and multivariate brain white matter differences in individuals with family history of Alzheimer's disease

**DOI:** 10.1002/alz.093909

**Published:** 2025-01-09

**Authors:** Stefanie A Tremblay, R. Nathan Spreng, Alfie Wearn, Zaki Alasmar, Amir Pirhadi, Christine L Tardif, M. Mallar Chakravarty, Sylvia Villeneuve, Ilana R Leppert, Felix Carbonell, Yasser Iturria Medina, Christopher J Steele, Claudine J Gauthier

**Affiliations:** ^1^ Concordia University, Montreal, QC Canada; ^2^ Montreal Neurological Institute, Montreal, QC Canada; ^3^ McGill University, Montreal, QC Canada; ^4^ StoP‐AD Centre, Douglas Mental Health Institute Research Centre, Montreal, QC Canada; ^5^ Montreal Neurological Institute, McGill University, Montréal, QC Canada; ^6^ Biospective, Inc., Montreal, QC Canada; ^7^ Ludmer Centre for Neuroinformatics and Mental Health, McGill University, Montreal, QC Canada; ^8^ Max Planck Institute for Human Cognitive and Brain Sciences, Leipzig, Saxony Germany; ^9^ Montreal Heart Institute, Montreal, QC Canada

## Abstract

**Background:**

Alzheimer’s disease (AD) is thought to result from a complex cascade of events involving several pathological processes. Recent studies have reported alterations in white matter (WM) microstructure in the early phase of AD, but WM remains understudied. We used a multivariate approach to capture the complexity and heterogeneity of WM pathologies and its links to cognition and AD risk factors in a more holistic manner.

**Method:**

The MRI data of 134 cognitively unimpaired older adults with a family history of AD from the PREVENT‐AD cohort were analysed. Diffusion‐weighted imaging and multi‐echo magnetization transfer, proton density and T1‐weighted data were used to compute several WM metrics (see Fig. 1b‐c). We used the Mahalanobis distance (D2) to summarize the MRI metrics into a single score, indicative of the degree a voxel’s microstructure differs relative to a reference. Voxel‐wise D2 was computed for each subject relative to the group average of all other subjects using the MVComp tool and D2 maps were residualized for age (Fig. 2). Partial Least Squares (PLS) analyses were then performed to relate WM D2 with cognition (RBANS) and AD risk factors, separately in each sex.

**Result:**

In males, there was only one significant latent variable (LV 1). There were extensive brain WM regions associated with this LV pattern: higher white matter D2, was associated with higher BMI, lower total cholesterol (likely due to lower HDL) and worse cognitive performance in all cognitive domains except attention (Fig. 2a‐b). In females, only the first LV was significant. Higher D2 in several WM tracts, including the inferior and superior longitudinal fasciculus, and the parahippocampal cingulum, was associated with lower education, and worse cognitive performance in all cognitive domains except attention and visuospatial construction. Higher WM D2 was also associated with several metabolic risk factors in females including higher SBP, higher BMI, higher glycated hemoglobin (HbA1c) and lower cholesterol (Fig. 2c‐d).

**Conclusion:**

The different patterns of associations observed suggest there are sex‐specific risk profiles associated with WM microstructure differences in this population of older adults at risk of AD. The WM tracts affected in each sex were also associated with specific cognitive profiles.